# Study of the *NOTCH3* Gene Reveals the First CADASIL Cases in Crete and a Novel Pathogenic Variant

**DOI:** 10.1002/brb3.70789

**Published:** 2025-09-25

**Authors:** Ioannis Zaganas, Ioannis Tsiverdis, Evgenia Kokosali, Ionela Litso, Minas Drakos, Irene Skoula, Alexandros Zabetakis, Lambros Mathioudakis, Vassilios Mastorodemos, Panayiotis Mitsias

**Affiliations:** ^1^ Neurology/Neurogenetics Laboratory, School of Medicine University of Crete Heraklion Crete Greece; ^2^ Department of Neurology University Hospital of Heraklion Heraklion Crete Greece; ^3^ Department of Neurology, School of Medicine University of Crete Heraklion Crete Greece; ^4^ Department of Neurology Henry Ford Hospital Detroit Michigan USA; ^5^ School of Medicine Wayne State University Detroit Michigan USA

**Keywords:** CADASIL, dementia, neurogenetics, *NOTCH3*, stroke

## Abstract

**Background:**

*NOTCH3* gene variants are associated with cerebral autosomal dominant arteriopathy with subcortical infarcts and leukoencephalopathy (CADASIL). In this study we aimed to examine the presence of pathogenic *NOTCH3* variants in individuals with suspected CADASIL on the Greek island of Crete. This represents the first report of CADASIL patients in Crete.

**Methods:**

We reviewed the medical records of the University Hospital of Heraklion and identified three patients with the clinical diagnosis of CADASIL. In these patients pathogenic NOTCH3 variants were identified through targeted or whole‐exome sequencing (WES).

**Results:**

A novel heterozygous variant in exon 4 of the *NOTCH3* gene (p.Cys206Trp; NM_000435.3:c.618C>G) was found in a 67‐year‐old woman who suffered from recurrent ischemic strokes, cognitive impairment, depression, and headache, as well as her son, who presented with headache, anxiety disorder, and insomnia. Brain MRI for both patients revealed white matter disease, including the anterior temporal lobes. The characteristics of this variant (a Cys‐related variant in the epidermal growth factor repeats area) support its pathogenicity. We also identified a 72‐year‐old patient affected by CADASIL and carrying a previously described p.Arg607Cys (NM_000435.3:c.1819C>T) *NOTCH3* variant.

**Conclusions:**

This report extends the geographic and genotypic spectrum of pathogenic NOTCH3 variants and documents the first CADASIL cases on the island of Crete, Greece.

## Graphical abstract

This study reports the first genetically confirmed cerebral autosomal dominant arteriopathy with subcortical infarcts and leukoencephalopathy (CADASIL) cases on the island of Crete, Greece. Medical record review at the University Hospital of Heraklion identified three patients with a clinical CADASIL diagnosis. Genetic analysis revealed a novel *NOTCH3* variant, p.(Cys206Trp), in a 67‐year‐old woman with stroke and cognitive and psychiatric symptoms and her son with milder features. Brain MRIs showed white matter lesions involving the anterior temporal lobes. A third patient, aged 72, carried the known pathogenic variant p.(Arg607Cys). These findings expand the geographic and mutational spectrum of CADASIL.

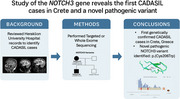

## 1 Introduction

Cerebral autosomal dominant arteriopathy with subcortical infarcts and leukoencephalopathy (CADASIL) is characterized by small‐vessel arteriopathy typically manifesting in early adulthood (20s–30s), leading to brain white matter damage (Chabriat et al. 2009; Yamamoto et al. 2023). The clinical manifestations of CADASIL include migraine, mood disorders, recurring strokes, transient ischemic attacks, and cognitive decline that progresses to dementia (Chabriat et al. 2009). On brain imaging, extensive white matter hyperintensities (WMH) of presumed vascular origin are found, which also affect the anterior temporal lobes, the external capsules, and, occasionally, the corpus callosum (O'Sullivan et al. 2001).

CADASIL is caused by pathogenic variants in the *NOTCH3* gene (Joutel et al. 1996). More than 280 such variants have been described (Yamamoto et al. 2023), with most of them clustering in exons 3 and 4 of the gene (Chabriat et al. 2020; Ni et al. 2022). The *NOTCH3* gene encodes the Notch3 protein, a type I transmembrane receptor expressed in vascular smooth muscle cells and pericytes (Hosseini‐Alghaderi and Baron 2020). The Notch3 protein has 34 epidermal growth factor repeats (EGFRs), each containing six cysteine residues that form stabilizing disulfide bonds. *NOTCH3* pathogenic variants are found throughout all EGFRs but most commonly cluster within repeats 1 to 6 and typically change the total number of cysteine residues of this area, either by introduction or elimination of a cysteine residue (Hosseini‐Alghaderi and Baron 2020). This in turn results in destabilization and misfolding of the extracellular part of the Notch3 receptor. The position of the *NOTCH3* variant could be associated with the age at onset of CADASIL and other clinical features (Xiromerisiou et al. 2020; Cho et al. 2022; Bugallo‐Casal et al. 2025; Gravesteijn et al. 2022). The estimated prevalence of CADASIL is 2–5/100,000 population, with phenotypic and genotypic characteristics differing among ethnic groups (Xiromerisiou et al. 2020; Kim et al. 2022). However, it is believed that it may be more common, as recent studies have shown that the prevalence of pathogenic *NOTCH3* variants in the general population could be as high as 3.4/1000 (Yamamoto et al. 2023; Wang 2018; Rutten et al. 2016). In Greece, a recent review identified 14 studies reporting 14 families diagnosed with CADASIL, with variants in the exon 4, p. Arg169Cys and p. Arg182Cys, being the most common finding, and no patients from Crete were included in these pooled analyses (Paraskevas et al. 2022). While arginine‐to‐cysteine substitutions are the most common in patients with CADASIL or CADASIL‐like cerebral small vessel disease, an East Asia‐specific substitution—arginine to proline (p.R75P)—was recently identified as the fifth most frequent variant (Boston et al. 2024).

In this study we aimed to identify the first CADASIL cases from Crete followed at the University Hospital of Heraklion, Crete, a referral center for complex stroke cases from Crete, the Aegean islands, and the continental country. Here we present two patients, members of the same family from Crete, Greece, where the use of next‐generation sequencing techniques combined with segregation analyses and in silico structural predictions identified a novel causative variant (p.Cys206Trp) in exon 4 of the *NOTCH3* gene, expanding the genotypic spectrum of CADASIL. In addition, we describe a third patient with CADASIL with another variant, already described in the literature (p.Arg607Cys). Thus, we extend the geographic distribution of CADASIL to the island of Crete beyond the cases from continental Greece described in previous studies.

## 2 Methods

### 2.1 Patient Consent and Ethical Guidelines

Informed consent was obtained from all individuals in this study. The study protocol was conducted following the ethical guidelines of the World Medical Association Declaration of Helsinki (version 2013) and approved by the Institutional Review Board of the University Hospital of Heraklion, Crete, Greece, as part of an extended genetic studies protocol.

### 2.2 Whole Exome Sequencing (Patient #1)

Since extensive diagnostic evaluation failed to reveal a definite etiology for the recurrent strokes of **Patient #1**, and a genetic origin was suspected, we proceeded to perform WES as the initial diagnostic approach.

Peripheral blood (∼5 mL) was collected from patient #1, and DNA extraction, WES, and initial bioinformatics analysis were performed at Macrogen Europe, Netherlands. Exome library preparation was performed using the Agilent V6 SureSelect Target Enrichment System. The DNA libraries were sequenced after exon enrichment on an Illumina NovaSeq 6000 platform with an estimated average coverage of 150X. The paired‐end sequences were mapped to the human reference genome hg38/GRCh38 to generate a BAM file that was recalibrated and used to call the variants with Haplotype Caller of Genome Analysis Toolkit (GATK). The called variants were subsequently filtered and annotated to generate VCF files.

Further annotation of the called variants was performed at the Neurology/Neurogenetics Laboratory, School of Medicine, University of Crete, using the Ingenuity Clinical Insight software (IVA, Qiagen, USA) and in‐house analysis methods, as previously described (Zaganas et al. 2021; Zaganas et al. 2020). The stepwise filtering included variants that produced a missense, nonsense, frameshift, in‐frame indel, or splice site change and excluded variants with minor allele frequency >1% on gnomAD and AFC (Allele Frequency Community). Assessing the functional consequences of the variants on encoded proteins was performed using prediction algorithms such as the Combined Annotation‐Dependent Depletion (CADD) score (Rentzsch et al. 2019), AlphaMissense (Cheng et al. 2023), and the Rare Exome Variant Ensemble Learner (Revel) (Ioannidis et al. 2016). Identified variants were further evaluated manually, taking into consideration the patient's phenotype, data from public databases, and available literature. The variant interpretation followed the 2015 guidelines of the American College of Medical Genetics (ACMG) (Richards et al. 2015).

### 2.3 Sanger Sequencing (Patients #1, #2, and #4 and Unaffected Son of Patient #1)

The two *NOTCH3* variants in exons 4 and 33 that were deemed possibly related to the patient's #1 phenotype were confirmed by Sanger sequencing at Diagenom (Rostock, Germany). Her two sons were similarly tested (patient #2 and the unaffected son). For patient #3, exons 2–6 and 11 were specifically sequenced. All sequences obtained were compared with the Ensembl transcript sequence ENST00000263388.

### 2.4 Protein Structure Prediction

AlphaFold Colab (Jumper et al. 2021) was used to predict the 3D structures of the fifth EGF‐like domain (residues 197–234) of the wild‐type and the p.Cys206Trp variant Notch3 protein, using the amino acid sequences as a template. The best models were selected based on the predicted local‐distance difference test (pLDDT) confidence values. The predicted structures were superimposed using the PyMOL Molecular Graphics System (Schrödinger, LLC) command “super.” The root‐mean‐square deviation (RMSD) values from the alignments were used to evaluate the differences between the two predicted structures.

## 3 Results

### 3.1 Patient #1

At the age of 58 years, the patient had an episode of aphasia lasting for several days. When evaluated 3 years later for language and memory problems, non‐specific headache, and depressive symptoms, cognitive testing revealed only mild impairment (MoCA test: 27/30). Five years after the initial episode, she experienced two more episodes of neurological dysfunction, one with dysarthria and one with left hemiparesis. Eight years after the first symptoms, examination revealed signs of cognitive decline (MoCA: 22/30, with deficits in visuospatial abilities, executive functioning, verbal fluency, and short‐term memory). One year later, the patient presented sudden‐onset aphasic disorder and gait difficulties due to paresis of the right lower extremity. After each one of the four episodes of acute neurological dysfunction, the patient recovered partially, and between these episodes, there was progressive deterioration of cognition. During the most recent examination, 10 years after the initial episode, she presented impairment in speech, spatial and temporal orientation, short‐term memory, and ability to calculate (MoCA: 19/30).

The patient had hyperlipidemia and hypertension and was a cigarette smoker. Her father died at the age of 100 years with no severe medical problems. Her mother died at the age of 68 years from breast cancer. One of her brothers died from cancer, and another one had depression from cirrhosis and “encephalitis.” Except for the two sons of patient #1, no other family member was available for clinical examination and testing.

**FIGURE 1:**
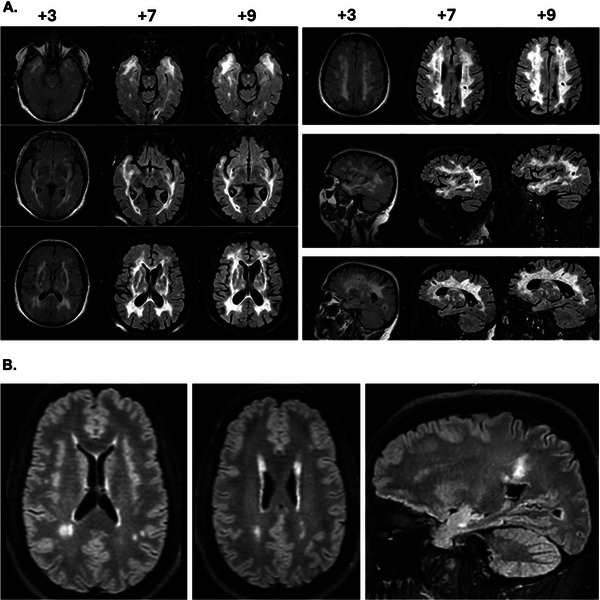
**(A)** Brain MRIs (FLAIR sequences, axial and sagittal sections) of the index patient (Patient #1) in different time periods (3, 7, and 9 years after the initial episode of neurological dysfunction). There is WMH of presumed vascular origin, which includes the anterior temporal lobes and external capsules, and evolves over time in consecutive MRI scans. The patient was found to harbor the p.Cys206Trp *NOTCH3* variant. **(B)** MRI (FLAIR sequences, axial and sagittal sections) of the index patient's son (Patient #2) carrying the p.Cys206Trp *NOTCH3* variant. A brain MRI scan showed WMH of presumed vascular origin, of similar nature but lesser extent than that of his mother (Patient #1). Abbreviations: WMH, White Matter Hyperintensities.

MRI of patient #1 demonstrated WMH of presumed vascular origin, which included the anterior temporal lobes and external capsules, but without evident spinal cord lesions (Figure 1Α). These changes progressively worsened over time, as observed in consecutive MRI scans (Figure 1Α). The patient underwent extensive diagnostic investigations to exclude causes of young‐onset stroke presenting as CNS small vessel disease or other leukoencephalopathies, including echocardiogram, cardiac rhythm monitoring, serum testing for thrombophilia and autoimmune disorders, and analysis for oligoclonal bands in the cerebrospinal fluid. She was prescribed antiplatelet and statin medications, with close monitoring of blood pressure, and she was strongly encouraged to quit smoking.

### 3.2 Patient #2

The son of patient #1, 33 years old, presented with chronic daily headaches (with migrainous features), anxiety disorder, and insomnia for years. Cognitive testing revealed no impairment. A brain MRI scan showed WMH of presumed vascular origin (Figure 1B), of a similar nature but lesser extent than that of his mother.

The second son of patient #1 did not complain of neurological symptoms, had a normal neurological examination, and had a normal brain MRI.

**FIGURE 2:**
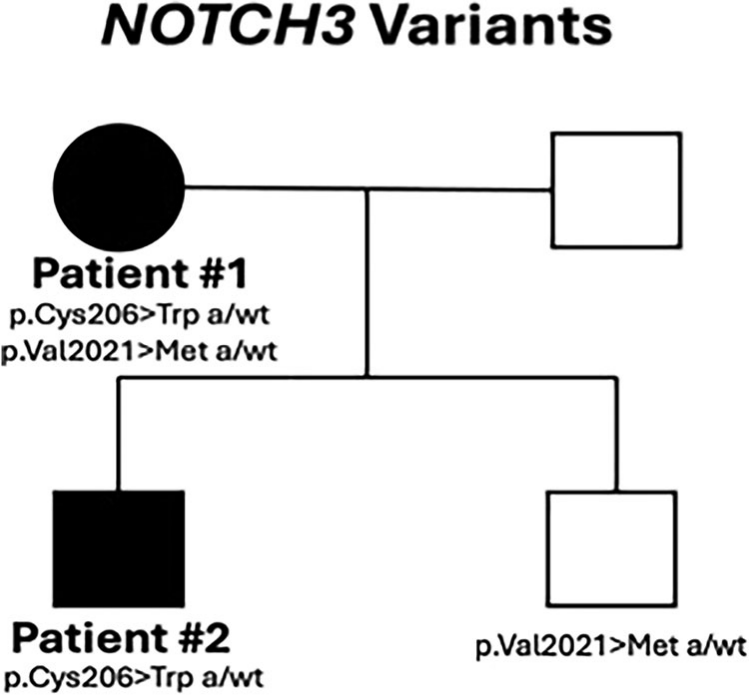
Pedigree of the family described here. Patients #1 and #2 are both heterozygous for the p.Cys206Trp *NOTCH3* variant, while the unaffected son is not a carrier of that variant. The p.Cys206Trp *NOTCH3* variant segregates with the pathogenic phenotype in this family. **Abbreviations**: a, alternative allele; wt, wild‐type allele.

**FIGURE 3:**
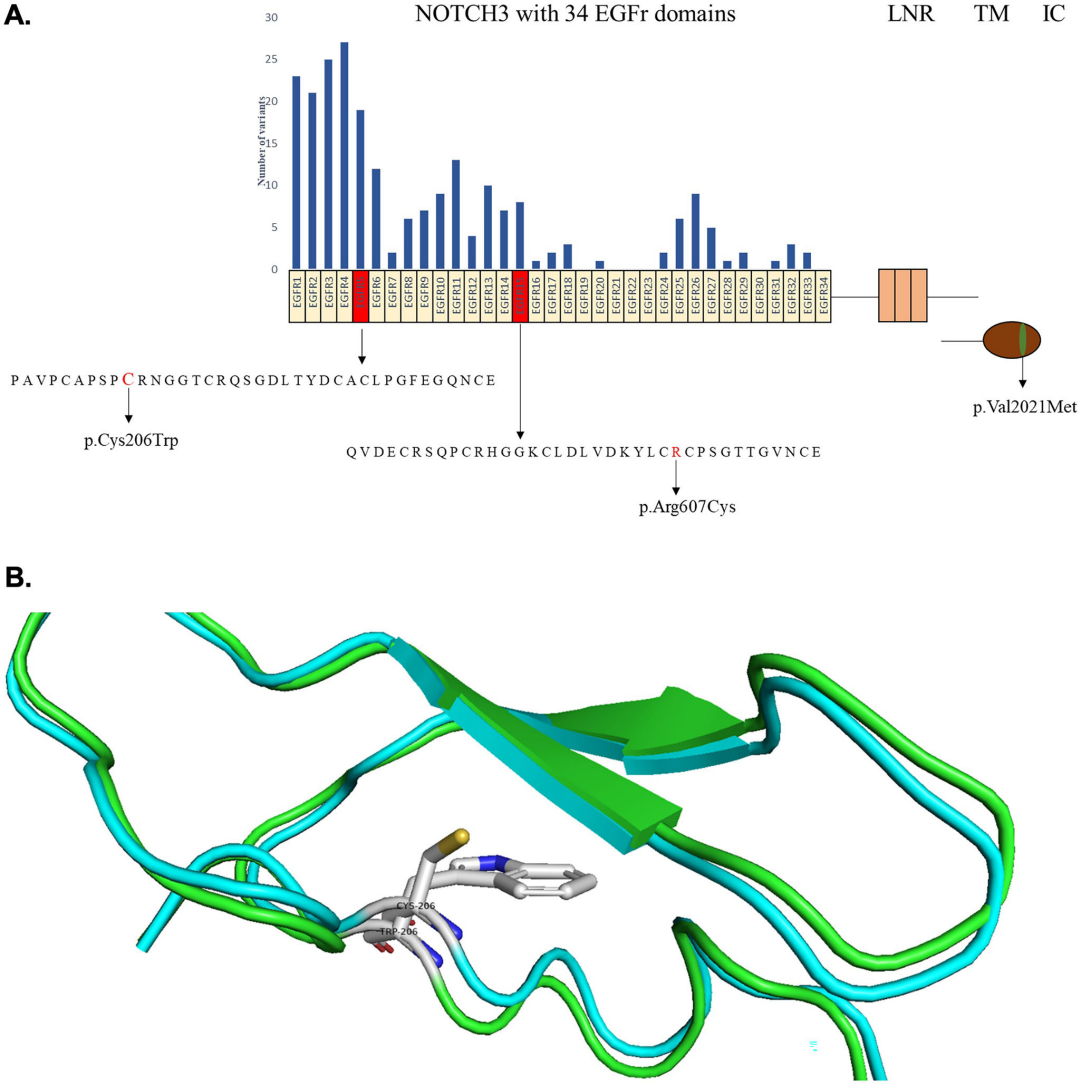
**(A)** Schematic representation of the Notch3 protein with the location of the p.Cys206Trp, the p.Arg607Cys, and other cysteine‐related variants described in the literature (Kang et al. 2021; Xiromerisiou et al. 2020; Cho et al., 2021). The Notch3 protein is a transmembrane protein composed of an extracellular, a transmembrane, and an intracellular domain. Each extracellular domain is composed of 34 EGFR domains and 3 LNR domains. The p.Cys206Trp variant is present in the fifth EGFr domain and the p.Arg607Cys is in the 15th EGFR domain, (shown in red). Also, a map of the *NOTCH3* pathogenic variants is presented, with columns representing the number of cysteine‐related variants in each EGFr domain. The p.Val2021Met variant is shown in green. **Abbreviations**: IC, intracellular domain; LNR: lin repeats; TM, transmembrane. **(B)** Superimposed structures of wild‐type (blue) and variant p.Cys206Trp (green) Notch3 proteins. The 3D structures represent the fifth EGF‐like domain (197‐234) of the Notch3 extracellular domain and were derived from AlphaFold Colab. (Cheng et al., 2023). The RMSD value between the two superimposed structures was estimated to be 0.755 Å. Even though the presence of the variant does not have a significant impact on the 3D structure of the protein, it is expected to affect interaction with the Notch3 ligand. The PyMOL Molecular Graphics System, Version 2.5, Schrödinger, LLC, was used to create the cartoons.

WES revealed two heterozygous genetic changes, p.Cys206Trp (NM_000435.3:c.618C>G) and p.Val2021Met (NM_000435.3:c.6061G>A), in exon 4 and exon 33, respectively, of the *NOTCH3* gene (Figure 2) as possibly causative variants for **Patient #1's** phenotype. Sanger sequencing confirmed that both variants were in a heterozygous state. Sanger sequencing of exons 4 and 33 of the *NOTCH3* gene for patient #1's affected son (**Patient #2**) revealed the presence of the p.Cys206Trp variant in a heterozygous state and the absence of the p.Val2021Met variant (Figure 2). In contrast, the clinically unaffected second son of Patient #1 did not harbor the p.Cys206Trp variant in the *NOTCH3* gene (Figure 2). Thus, the p.Cys206Trp *NOTCH3* gene variant segregated with the CADASIL phenotype in this family, as it was detected only in the two affected family members (Figure 2). Specifically, by employing the ACMG 2015 Guidelines, p.Cys206Trp was classified as likely pathogenic (PP3, PM1, PM2_SUP, PM5). Finally, at the structural level, using AlphaFold‐derived 3D structures of the EGF‐like domain of the Notch3 extracellular domain (Figure 3Β), we estimated an RMSD value of 0.755 Å between the two superimposed structures (harboring a Cys or Trp residue at 206). Even though this RMSD value change is small, this amino acid change is expected to interfere with the stability of the cysteine‐rich region (by altering the total number of cysteine residues) and to affect interaction with the Notch3 ligands, as shown for other pathogenic *NOTCH3* variants (Papakonstantinou et al. 2019).

### 3.3 Patient #3

Through a systematic search of the medical records of the University Hospital of Heraklion, we identified an additional patient harboring the p.Arg607Cys (NM_000435.3:c.1819C>T) *NOTCH3* variant, which has been repeatedly reported as pathogenic (Qin et al. 2019; Jokumsen‐Cabral et al. 2019). This 72‐year‐old patient, unrelated to the three individuals described previously, presented with multiple ischemic infarcts (one of them treated with thrombolysis), cognitive impairment, gait unsteadiness, head tremor, migraine for at least 15 years, and WMH of presumed vascular origin in consecutive brain MRIs.

## 4 Discussion

In order to examine the presence of CADASIL cases in Crete, we report a family with two affected members harboring the novel p.Cys206Trp variant in exon 4 of the *NOTCH3* gene, further expanding the genotypic spectrum of pathogenic *NOTCH3* variants. Taking into account another Cretan patient affected with CADASIL and harboring the already described p.Arg607Cys *NOTCH3* variant, we describe a total of three patients with CADASIL from Crete, an area of Greece not included in previous related studies (Paraskevas et al. 2022).

Although the p.Cys206Trp change has not been described in the literature, it is classified as likely pathogenic based on ACMG 2015 guidelines. Specifically, apart from being co‐inherited with the disease in the currently reported family (Figure 2), it is not found in population databases such as gnomAD. It localizes in a functional region of the protein, where no benign changes are detected (Figure 3Α). Of note, the p.Cys206Trp variant resides in exon 4 of the *NOTCH3* gene, where most CADASIL‐associated variants have been found in Greek and other populations (Paraskevas et al. 2022). In support of the possible pathogenicity of the p.Cys206Trp variant, there are large differences in physicochemical properties between cysteine ​​and tryptophan, and this change is predicted to be possibly harmful according to AlpaMissense (score = 0.996). Importantly, alternative variants at the same position, specifically p.Cys206Tyr (Matsumoto et al. 2005) and p.Cys206Arg (Tikka et al. 2009), have been described to lead to CADASIL.

In contrast, there are conflicting criteria for the pathogenicity of the p.Val2021Met *NOTCH3* gene change. It does not segregate with the disease in the family described here and has a greater frequency in the population than expected for CADASIL. Thus, despite being in a relatively evolutionarily conserved amino acid site (PhyloP100way = 7.622) and having been described before in the literature as possibly involved in AD pathogenesis (Nicolas et al. 2016; Sassi et al. 2018; Alanis‐Funes et al. 2022), it is apparently not the cause of CADASIL in this family.

Concerning the clinical presentation of the three patients described here, small vessel disease‐related ischemic strokes, cognitive impairment, depression, and headache have been frequently described previously, both in Greece and worldwide (Cho et al. 2022; Paraskevas et al. 2022; Bersano et al. 2018). On MR imaging, white matter hyperintensities were present in the anterior temporal lobe, the subcortical white matter, and the external capsule in our index patient (Patient #1), whereas in her son (Patient #2), only the subcortical white matter and the right anterior temporal lobe were affected. No microbleeds were evident in the MRI scans of both patients. These imaging findings are compatible with those reported previously (Cho et al. 2022; Paraskevas et al. 2022; Bersano et al. 2018; Viswanathan et al. 2007).

Given the globally observed estimated minimum prevalence of 2–5 CADASIL cases per 100,000 (Rutten et al. 2016), for Crete (population of approximately 650,000), we expect 12–30 cases, a number much higher than the three cases reported here. In this respect, it is common for CADASIL patients to be misdiagnosed initially with sporadic small vessel disease, multiple sclerosis, or other leukoencephalopathies. Of note, there are reports based on massive sequencing data of large cohorts that show a general population prevalence (per 100,000) of pathogenic (cysteine‐altering) *NOTCH3* variants as high as 220 in the United Kingdom (Cho et al. 2021), 340 in the exome aggregation consortium (ExAC) database (Rutten et al. 2016), 440 in Korea (Kang et al. 2021), and 900 in Taiwan (Lee et al. 2020).

The novel p.Cys206Trp *NOTCH3* change described here extends the genotypic spectrum of CADASIL pathogenic variants. In addition, the two patients harboring this novel variant, as well as a third patient with the p.Arg607Cys *NOTCH3* variant already described in the literature, come from an area of Greece (Crete), where no CADASIL cases have been published before, extending the geographic spectrum of the disease.

## Author Contributions

**Ioannis Zaganas**: conceptualization, data curation, funding acquisition, formal analysis, methodology, investigation, project administration, resources, supervision, validation, writing–original draft, writing–review and editing. **Ioannis Tsiverdis**: writing–review and editing and investigation. **Evgenia Kokosali**: formal analysis, investigation, methodology, visualization, writing–review and editing. **Ionela Litso**: formal analysis, methodology, software, visualization, writing–review and editing. **Minas Drakos**: formal analysis, visualization, writing–review and editing. **Irene Skoula**: investigation, project administration, writing–review and editing. **Alexandros Zabetakis**: investigation, writing–review and editing. **Lambros Mathioudakis**: investigation, writing–review and editing, and formal analysis. **Vassilios Mastorodemos**: investigation, writing–review and editing. **Panayiotis Mitsias**: supervision, writing–review and editing.

## Conflicts of Interest

The authors declare no conflicts of interest.

## Ethics Statement

The study protocol was conducted following the ethical guidelines of the World Medical Association Declaration of Helsinki (version 2013) and approved by the Institutional Review Board of the University Hospital of Heraklion, Crete, Greece, as part of an extended genetic studies protocol.

## Consent

Written informed consent was obtained from all individuals in this study.

## Peer Review

The peer review history for this article is available at https://publons.com/publon/10.1002/brb3.70789.

## Data Availability

WES and imaging data from the patients described here are available upon reasonable request by qualified researchers.
